# RNA-Seq Analysis of the Effect of Kanamycin and the ABC Transporter AtWBC19 on *Arabidopsis thaliana* Seedlings Reveals Changes in Metal Content

**DOI:** 10.1371/journal.pone.0109310

**Published:** 2014-10-13

**Authors:** Ayalew Mentewab, Kinnari Matheson, Morayo Adebiyi, Shanice Robinson, Brianna Elston

**Affiliations:** 1 Biology Department, Spelman College, Atlanta, Georgia, United States of America; 2 Molecular Biology Department, Princeton University, Princeton, New Jersey, United States of America; 3 The Graduate School of Biomedical Sciences, University of Texas Health Science Center at Houston, Houston, Texas, United States of America; 4 College of Health Care Sciences, Nova Southeastern University, Davie, Florida, United States of America; Louisiana State University and A & M College, United States of America

## Abstract

Plants are exposed to antibiotics produced by soil microorganisms, but little is known about their responses at the transcriptional level. Likewise, few endogenous mechanisms of antibiotic resistance have been reported. The *Arabidopsis thaliana* ATP Binding Cassette (ABC) transporter AtWBC19 (ABCG19) is known to confer kanamycin resistance, but the exact mechanism of resistance is not well understood. Here we examined the transcriptomes of control seedlings and *wbc19* mutant seedlings using RNA-seq analysis. Exposure to kanamycin indicated changes in the organization of the photosynthetic apparatus, metabolic fluxes and metal uptake. Elemental analysis showed a 60% and 80% reduction of iron uptake in control and *wbc19* mutant seedlings respectively, upon exposure to kanamycin. The drop in iron content was accompanied by the upregulation of the gene encoding for FERRIC REDUCTION OXIDASE 6 (FRO6) in mutant seedlings but not by the differential expression of other transport genes known to be induced by iron deficiency. In addition, *wbc19* mutants displayed a distinct expression profile in the absence of kanamycin. Most notably the expression of several zinc ion binding proteins, including ZINC TRANSPORTER 1 PRECURSOR (ZIP1) was increased, suggesting abnormal zinc uptake. Elemental analysis confirmed a 50% decrease of zinc content in *wbc19* mutants. Thus, the antibiotic resistance gene WBC19 appears to also have a role in zinc uptake.

## Introduction

Plants are exposed to a variety of antibiotics produced by soil microorganisms. One such antibiotic is kanamycin, originally isolated from the soil bacterium *Streptomyces kanamyceticus*. Many plant species display exquisite sensitivity to a number of antibiotics, hence their usefulness as selection agents in plant genetic engineering. Kanamycin belongs to the aminoglycoside class of antibiotics. Aminoglycosides are multifunctional hydrophilic sugars that possess several amino and hydroxy groups. Aminoglycosides directly bind to the A site of the 16S rRNA of prokaryotic ribosomes causing codon misreading and inhibiting translocation [1], [2], [3]. The presence of an adenosine at position 1408 of the 16S rRNA is a key feature of prokaryotic ribosomes that allows binding of aminoglycosides. In the eukaryotic rRNA, the corresponding position is occupied by a guanine resulting in a lowered affinity for aminoglycosides [4]. Based on the similarity of their ribosomes to those of prokaryotes, plastids and mitochondria may represent the primary targets of aminoglycoside antibiotics in plants.

Plant responses to antibiotics have been poorly characterized. Nonetheless, several observations support the notion that chloroplasts are a major target of aminoglycosides. When plants are exposed to kanamycin, a characteristic symptom is the bleaching of the leaves indicating the breakdown of cholorophyll in choloroplasts [5]. When the chloroplast transporter MAR1 is mutated [6] or silenced [7], plants become resistant to kanamycin and several other aminoglycosides. MAR1 has a role in iron homeostasis and is thought to allow the passage of aminoglycosides into the chloroplast given their structural resemblance with chelated iron molecules [8].

Despite their sensitivity, *Arabidopsis* plants harbor at least one gene that confers antibiotic resistance – that encoding for the ABC transporter WBC19 (ABCG19). ABC transporters are defined by the presence of the highly conserved ATP Binding Cassette (ABC) domain responsible for the hydrolysis of ATP and a transmembrane domain (TMD) responsible for solute movement across the phospholipid bilayer. With a single ABC domain and a single TMD domain encompassing six predicted alpha helices, WBC19 is categorized as a half ABC molecule. Such half ABC molecules are thought to dimerize to form functional transporters. When WBC19 is mutated, kanamycin sensitivity of seedlings is exacerbated while expression of WBC19 in tobacco resulted in increased levels of antibiotic resistance [9]. In tobacco (*Nicotiana tabacum*), WBC19 was found to be specific to kanamycin, but in hybrid aspen (*Populus canescens x P. grandidentata*) it also showed resistance to three other aminoglycoside antibiotics - neomycin, geneticin, and paromomycin [10]. Further investigation in bacteria showed WBC19 did not confer resistance to kanamycin in *E. coli* [11]. A direct role of WBC19 in transport was not investigated as it was not localized in a membrane compartment. Thus the mechanism of antibiotic resistance associated with WBC19 has not been fully characterized.

The presence of several hydroxyl and amine groups makes kanamycin a polar compound. As a result, it poorly diffuses through membranes. Before reaching chloroplasts it has to first enter root cells, get mobilized through the vascular system and unloaded - most likely by co-opting other transporters. Given the previously reported association between antibiotic resistance and metal transport [6], [8], we postulate that endogenous mechanisms of antibiotic resistance, specifically the one associated with WBC19 is also linked to the transport of metals. Here we examined the transcriptomes and analyzed the metal content of control and *wbc19* mutant seedlings. We report that kanamycin induces transcriptional changes revealing an adaptation of the photosynthetic apparatus, metabolic fluxes and metal uptake. Most significantly, the iron content of seedling exposed to kanamycin is drastically reduced and the zinc content of *wbc19* mutants is lower even under normal conditions. Overall, our findings suggest that the antibiotic resistance gene WBC19 has a role in zinc homeostasis and to a lesser extent that of the other metals examined, copper and manganese.

## Materials and Methods

### RNA-seq transcriptome analysis

#### Plant Material

Control (SALK_064816C) and *wbc19* mutant (SALK_107731) lines generated by Alonso et al. [12] were previously obtained from the Arabidopsis Biological Resource Center (Columbus, OH). Homozygous seeds were plated under sterile conditions on MS media with or without kanamycin 50 mg/l. SALK_064816C mutants served as controls as they carry a T-DNA insertion that does not disrupt a gene. The T-DNA insertion is located in the vicinity of the TRANSPARENT TESTA 4 gene, 260 bp upstream of the start codon and the mutants have no visible phenotype. After stratification (3 days at 4°C), plates were transferred to a controlled-environment cabinet (23°C, 16 h light; 18°C, 8 h dark) and plant material was harvested and flash frozen 5 days later.

#### Library Preparation

Total RNA was extracted using a Plant RNeasy Mini Kit (Qiagen) and poly(A) mRNA isolated using oligo(dT) DynaBeads (Life Technologies). Messenger RNA fragmentation and RNA-seq library generation was according to [13]. Briefly, after alkaline fragmentation of mRNA, ∼25–45 mer fragments were gel purified on a 10% denaturing polyacrylamide-urea gel. After 3′-dephosphorylation with polynucleotide kinase (PNK, New England Biolabs) a 3′ adaptor was ligated to the fragments. Following a second gel purification step, the fragments were 5′phosphorylated with PNK and the 5′ adapter ligated. Each library was then amplified for 21 cycles and gel purified on a 90% formamide, 8% acrylamide denaturing gel. Four libraries, one per treatment condition were submitted for single-end sequencing on the Illumina GAII high throughput sequencing platform.

#### Analysis pipeline

Sequence analysis was carried out in the iPlant Collaborative Discovery Environment (www.iplantcollaborative.org). Reads were processed with FASTX clipper (version 0.0.6) to remove adapter sequences and keep sequences of 36 mer. Processed reads were aligned to the *Arabidopsis* thaliana reference genome (TAIR10) using TopHat2 (version 2.0.5) [14], [15]. The minimum read length was set to 18 and up to 2 mismatches were tolerated. Mapped reads were assembled and merged using Cufflinks (version 2.0.2) and Cuffmerge (version 2.0.2) respectively [16]. Transcript abundance and differential expression was analyzed using Cuffdiff (version 1.3.0) [16] whenever a minimum of 10 reads were available. Differential gene expression across samples was deemed significant when expression differences reached 2 fold and p<0.001. Gene descriptions and GO classifications were obtained from The Arabidopsis Information Resource (TAIR). The Functional Classification SuperViewer tool (http://bar.utoronto.ca/ntools/cgi-bin/ntools_classification_superviewer.cgi) was used to identify gene groups that were significantly over-represented under each treatment condition compared with their relative amount in the whole genome. GO Classifications were further mined for the words photosynthesis, oxidoreduction, electron carrier and various metals to identify specific transcripts among those that were differentially expressed.

### Real-Time PCR

Two sets of Real-Time PCR assays were performed. To validate the RNA-seq results, a set of 11 genes were randomly selected among those with the GO classifications of interest as listed in **Tables 1** **–** **6**. The second set of genes consisted of a panel of 5 iron homeostasis genes. Both sets of genes are listed in **Table S1**. To perform Real-Time PCRs, homozygous *wbc19* mutants (SALK_107731) and controls (SALK_064816C) seedlings were grown with or without kanamycin as above, RNA was extracted using the Plant RNeasy Mini Kit (Qiagen) and treated with DNAseI (Life Technologies). The cDNA was then synthesized using the SuperScript VILO cDNA synthesis kit (Life Technologies). Samples were diluted 200 fold before use for real-time assays. The TaqMan Universal Master Mix II with Uracil-N-Glycosylase (Life Technologies) was used along with predesigned TaqMan Assays (Life Technologies) as listed in **Table S1**. All selected assays featured probes that span exons. Target genes were FAM labeled whereas the control RAD23C was labeled with VIC for use in multiplex reactions. For each gene, Real-Time PCR assays were performed with samples representing all 4 experimental conditions and included 4 biological replicates and 3 technical replicates. Data was analyzed using the Pfaffl method [18]; comparisons between experimental conditions were carried out using a Student T-test and deemed significant when P<0.05.

**Table 1:** Differentially expressed transcripts encoding for proteins involved in photosynthesis and oxidoreduction upon seedling germination on kanamycin containing media.

GO ID	Description	Gene ID	Description	control rpkm	Control + kan rpkm	log2 (fold change)
GO:0015979	photosynthesis	AT2G46820	PHOTOSYSTEM I P SUBUNIT (PSI-P)	339.20	732.37	1.11
GO:0015979	photosynthesis	AT1G29920	CHLOROPHYLL A/B-BINDING PROTEIN 2(CAB2) (LHCB1.1)	122.61	979.94	2.99
GO:0016491	oxidoreductase activity	AT5G08740	NAD(P)H dehydrogenase C1 (NDC1)	22.47	0.34	−6.01
GO:0016491	oxidoreductase activity	AT4G32360	Mitochondrial Ferredoxin reductase(AtMFDR)	0.63	24.83	5.29
GO:0016491	oxidoreductase activity	AT2G40370	LACCASE 5 (LAC5) *	0.81	28.42	5.12
GO:0016491	oxidoreductase activity	AT5G66920	SKU5 similar 17 (sks17) *	4.21	75.98	4.17
GO:0016491	oxidoreductase activity	AT2G37240	Thioredoxin superfamily protein	25.53	0.57	−5.48
GO:0015035	protein disulfideoxidoreductaseactivity	AT5G18600	Thioredoxin superfamily protein	18.50	207.63	3.48

The terms photosynthesis and oxidoreduction were searched in the GO IDs associated with genes differentially expressed when comparing transcripts from control seedlings germinating on MS media to transcripts from control seedlings germinating on MS media with 50 mg/l kanamycin (minimum 2 fold difference in expression, P<0.001). * denotes genes also identified as encoding for metal binding proteins in **Table 2**.

**Table 2:** Transcripts encoding for metal binding proteins differentially expressed upon seedling germination on kanamycin containing media.

GO ID	Description	GeneID	Description	controlrpkm	control +kan rpkm	log2 (foldchange)
GO:0008270	zinc ionbinding	AT2G29580	MOS4-ASSOCIATEDCOMPLEX SUBUNIT5B (MAC5B)	31.66	0.35	−6.49
GO:0008270	zinc ionbinding	AT1G09920	TRAF-type zincfinger protein	2.57	100.45	5.28
GO:0004222	Metalloendopeptidase	AT5G65620	Zincin-likemetalloprotease	20.76	0.25	−6.32
GO:0005507	copper ionbinding	AT2G36950	Heavy metaltransport/detoxificationsuperfamilyprotein	24.80	0.37	−6.05
GO:0005507	copper ionbinding	AT4G33680	L,L-diaminopimelateaminotransferase	54.00	1.29	−5.37
GO:0005507	copper ionbinding	AT5G66920	SKU5 SIMILAR17 (sks17) *	4.21	75.98	4.17
GO:0005507	copper ionbinding	AT2G40370	LACCASE5 (LAC5) *	0.81	28.42	5.12
GO:0005507	copper ionbinding	AT5G55200	MITOCHONDRIALGRPE 1 (MGE1)	1.03	71.28	6.10
GO:0015095	magnesium iontransmembranetransporteractivity	AT3G19640	MAGNESIUMTRANSPORTER4 (MGT4)	0.70	28.06	5.32
GO:0030151	molybdenum ionbinding	AT1G30910	Molybdenumcofactor sulfurasefamily protein	1.17	53.01	5.49

The terms metal, iron, zinc, copper, manganese, magnesium, cobalt, molybdenum etc were searched in the GO terms associated with genes differentially expressed when comparing transcripts from control seedlings germinating on MS media to transcripts from control seedlings germinating on MS media with 50 mg/l kanamycin (minimum 2 fold difference in expression, P<0.001). * denotes genes also identified as encoding for oxidoreductases in **Table 1** **.**

**Table 3:** Transcripts encoding for proteins involved in photosynthesis and oxidoreduction differentially expressed in *wbc19* mutant seedlings.

GOID	Description	GeneID	Description	controlrpkm	mutantrpkm	log2 (foldchange)
GO:0015979	photosynthesis	AT1G76450	Photosystem IIreaction centerPsbP family protein	50.43	0.83	−5.92
GO:0015979	photosynthesis	AT3G05410	Photosystem IIreaction center PsbPfamily protein	1.29	128.25	6.63
GO:0015979	photosynthesis	AT3G53920	RNAPOLYMERASESIGMA-SUBUNIT C(SIGC)	3.54	82.28	4.54
GO:0015979	photosynthesis	AT2G18790	PHYTOCHROME B (PHYB)	14.70	0.19	−6.27
GO:0009055	electron carrieractivity	AT2G27510	FERREDOXIN3 (FD3) *	75.02	1.11	−6.08
GO:0016491	oxidoreductaseactivity	AT1G34200	Glyceraldehyde-3-phosphate dehydrogenase-like family protein	0.83	85.45	6.68
GO:0015035	protein disulfideoxidoreductaseactivity	AT2G32920	ARABIDOPSISTHALIANA PROTEINDISULFIDE ISOMERASE9 (ATPDI9)	30.76	0.48	−6.02
GO:0016705	oxidoreductase activity,acting on paired donors,with incorporationor reduction ofmolecularoxygen	AT3G14650	CYTOCHROME P450,FAMILY 72, SUBFAMILYA, POLYPEPTIDE 11”(CYP72A11) *	19.93	0.36	−5.77

The terms photosynthesis, oxidoreduction and electron carrier were searched in the GO associated with genes differentially expressed when comparing transcripts from control seedlings to transcripts from *wbc19* mutant seedlings germinating on MS media (minimum 2 fold difference in expression; P<0.001). * denotes genes also identified as encoding for metal binding proteins in **Table 4** **.**

**Table 4:** Transcripts encoding for metal binding proteins differentially expressed in *wbc19* mutant seedlings.

GOID	Description	GeneID	Description	controlrpkm	mutantrpkm	log2 (foldchange)
GO:0008270	zinc ionbinding	AT5G59820	RESPONSIVE TOHIGH LIGHT 41(RHL41)	6.28	197.89	4.98
GO:0008270	zinc ionbinding	AT4G21350	PLANT U-BOX8 (PUB8)	2.23	110.71	5.63
GO:0008270	zinc ionbinding	AT5G01450	RING/U-boxsuperfamily protein	0.71	45.47	6.00
GO:0008270	zinc ionbinding	AT1G68190	B-box zinc fingerfamily protein	1.00	107.51	6.74
GO:0008270	zinc ionbinding	AT3G46090	C2H2-type zinc fingerprotein ZAT7	1.88	211.01	6.81
GO:0006829	zinc iontransport	AT3G12750	ZINC TRANSPORTER1 PRECURSOR (ZIP1)	0.91	100.73	6.79
GO:0005506	iron ionbinding	AT3G14650	CYTOCHROME P450,FAMILY 72, SUBFAMILYA, POLYPEPTIDE 11 (CYP72A11) *	19.93	0.36	−5.77
GO:0051536	iron-sulfurclusterbinding	AT2G27510	FERREDOXIN 3(FD3) *	75.02	1.10	−6.08

The terms metal, iron, zinc, copper, manganese, magnesium, cobalt, molybdenum etc were searched in the GO terms associated with genes differentially expressed when comparing transcripts from control seedlings to transcripts from *wbc19* mutant seedlings germinating on MS media (minimum 2 fold difference in expression; P<0.001). * denotes genes also identified as encoding for oxidoreductases in **Table 3** **.**

**Table 5:** Transcripts encoding for proteins involved in photosynthesis and oxidoreduction differentially expressed when comparing control and *wbc19* mutant seedlings geminating on MS media with 50 mg/l kanamycin.

GOID	Description	GeneID	Description	Control+ kanrpkm	Mutant+ kanrpkm	log2 (foldchange)
GO:0015979	photosynthesis	AT1G29920	CHLOROPHYLLA/B-BINDINGPROTEIN 2 (CAB2)(LHCB1.1)	979.95	0.01	−16.58
GO:0015979	photosynthesis	ATCG00270	PHOTOSYSTEM IIREACTION CENTERPROTEIN D (PSBD)	1000.97	334.95	−1.58
GO:0015979	photosynthesis	AT1G74880	NADH DEHYDROGENASE-LIKE COMPLEX)(NdhO)	5.85	113.43	4.28
GO:0009055	electron carrieractivity	AT5G49730	FERRIC REDUCTIONOXIDASE 6 (FRO6)	7.18	75.65	3.40
GO:0009055	electron carrieractivity	AT5G08740	NAD(P)H DEHYDROGENASEC1 (NDC1)	0.35	16.69	5.58
GO:0009055	electron carrieractivity	AT1G06820	CAROTENOID ISOMERASE(CRTISO)	1.34	36.41	4.76
GO:0009055	electron carrieractivity	AT1G64640	EARLY NODULIN-LIKEPROTEIN 8 (ENODL8)	97.44	2.04	−5.58
GO:0009055	electron carrieractivity	AT2G47470	UNFERTILIZED EMBRYOSAC 5 (UNE5)	6.31	85.77	3.77
GO:0016491	oxidoreductaseactivity	AT2G37240	Thioredoxin superfamilyprotein	0.57	28.75	5.65
GO:0016616	oxidoreductase activity,acting on the CH-OHgroup of donors, NADor NADP as acceptor	AT4G29010	ABNORMAL INFLORESCENCEMERISTEM (AIM1)	7.05	61.77	3.13
GO:0016627	oxidoreductase activity,acting on the CH-CHgroup of donors	AT5G65110	ACYL-COA OXIDASE 2(ACX2)	1.49	36.79	4.62
GO:0016639	oxidoreductase activity,acting on the CH-NH2group of donors,NAD or NADPas acceptor	AT5G07440	GLUTAMATEDEHYDROGENASE 2(GDH2)	147.51	39.11	−1.92

The terms photosynthesis, oxidoreduction and electron carrier were searched in the GO IDs associated with genes differentially expressed when comparing transcripts from control and *wbc19* mutant seedlings germinating on MS media with 50 mg/l of kanamycin (minimum 2 fold difference in expression; P<0.001).

**Table 6:** Transcripts encoding for metal binding proteins differentially expressed when comparing control and *wbc19* mutant seedlings geminating on MS media with 50 m/l kanamycin.

GO ID	Description	GeneID	Description	control+ kanrpkm	Mutant+ kanrpkm	log2 (foldchange)
GO:0000041	transition metalion transport	AT2G36290	alpha/beta-Hydrolasessuperfamily protein	49.93	0.94	−5.73
GO:0005506	iron ion binding	AT5G49730	FERRIC REDUCTIONOXIDASE 6 (FRO6)	7.18	75.65	3.4
GO:0071281	cellular responseto iron ion	AT5G04950	NICOTIANAMINESYNTHASE 1 (NAS1)	46.06	1.24	−5.22
GO:0005507	copper ionbinding	AT2G36950	Heavy metal transport/detoxification superfamily	0.37	17.67	5.56
GO:0005507	copper ionbinding	AT5G07440	GLUTAMATEDEHYDROGENASE 2(GDH2)	147.51	39.11	−1.92
GO:0005507	copper ionbinding	AT5G55200	MITOCHONDRIALGRPE 1 (MGE1)	71.29	2.15	−5.05
GO:0008270	zinc ionbinding	AT1G76950	(PRAF1)	31.11	2.03	−3.94
GO:0008270	zinc ionbinding	AT3G42790	ALFIN-LIKE 3(AL3)	45.02	1.18	−5.26
GO:0008270	zinc ionbinding	AT3G43240	ARID/BRIGHT DNA-binding domain-containingprotein	0.25	13.69	5.78
GO:0008270	zinc ionbinding	AT5G48250	B-box type zinc fingerprotein with CCT domain	33.25	0.77	−5.44
GO:0004222	metalloendopeptidaseactivity	AT5G65620	Zincin-like metalloproteasesfamily protein	0.26	35.28	7.09
GO:0008237	metallopeptidaseactivity	AT4G20070	ALLANTOATEAMIDOHYDROLASE(AAH)	0.35	19.08	5.77
GO:0015095	magnesiumion transmembranetransporter activity	AT3G19640	MAGNESIUMTRANSPORTER4 (MGT4)	28.06	0.72	−5.29
GO:0070838	divalent metalion transport	AT4G23890	NADH DEHYDROGENASE-LIKE COMPLEX S(NdhS)	45.4	1.21	−5.23
GO:0051539	4 iron, 4 sulfurcluster binding	AT5G04560	DEMETER(DME)	22.58	1.34	−4.08
GO:0016226	iron-sulfurcluster assembly	AT1G14030	LYSINE METHYLTRANSFERASE(LSMT)-LIKE (LSMT-L)	51.02	1.57	−5.03
GO:0030145	manganeseion binding	AT4G29490	Metallopeptidase M24family protein	54.69	1.56	−5.13
GO:0046872	metal ionbinding	AT1G65290	MITOCHONDRIAL ACYLCARRIER PROTEIN 2 (mtACP2)	2.01	90.61	5.49
GO:0046872	metal ionbinding	AT4G00900	ER-TYPE CA2+-ATPASE2 (ECA2)	21.46	0.4	−5.75
GO:0010038	response tometal ion	AT5G26240	CHLORIDE CHANNELD (CLC-D)	0.25	19.85	6.34

### Metal Analysis

To evaluate the effect of kanamycin, 5 day old seedlings from homozygous *wbc19* mutants (SALK_107731) and controls (SALK_064816C) were grown on media with or without kanamycin 50 mg/l as above. Seedlings were collected from plates and rinsed 3 times with 18 mΩ distilled water and dried for 2 d at 70°C. The plant material was weighed before shipping to the Lab for Environmental Analysis at the University of Georgia, Athens (UGA) where samples were digested with nitric acid before metal analysis by Inductively Coupled Plasma- Mass Spectrometry (ICP-MS) for Fe, Zn, Mn and Cu. For each treatment condition, metal content was analyzed using 3 biological replicates.

### Expression pattern of WBC19

The promoter region of WBC19 (2130 bp upstream of the start codon) was amplified from genomic DNA extracts of WT Arabidopsis plants using the forward 5′ CACCTTTGGGACCCCCAAAATCTC 3′ and reverse 5′ TCCCGGACGGCGTCGTTTAGA 3′ primers. The resulting fragment was cloned in the pENTR-D-TOPO vector (LifeTechnologies) and checked by restriction digestion. It was subsequently recombined with the pMDC162 vector designed for the expression of promoter-β-glucuronidase (GUS) fusions [19] and again checked by restriction digestion.

WT Arabidopsis plants were transformed with *Agrobacterium tumefaciens* strain GV3850 containing the construct using the floral dip method [20]. Seven transgenic plants were recovered and grown to maturity. Seeds were collected, sterilized and germinated on MS media and plants were transferred to soil after 2 weeks. Plant material was collected throughout development, fixed in 90% cold acetone for 20 minutes on ice and stained for histochemical localization of GUS activity [21].

### Accession number

The RNA-seq data used in this study is available in the Gene Expression Omnibus(GEO) database under accession number GSE58662.

## Results

RNA-seq analysis was conducted to capture the effect of kanamycin on *Arabidopsis* seedlings and examine how the response was modulated by WBC19. Control and *wbc19* mutant seedlings were grown with or without 50 mg/l kanamycin. The *wbc19* mutants used were T-DNA insertional knockouts in which the bacterial kanamycin resistance gene, neomycin phosphotransferase type II (NPTII) is expressed at a relatively high level [9]. Yet, because of the disruption of *wbc19* they are sensitive to kanamycin at 50 mg/l. To compensate for the confounding effect that might be added by the NPTII gene, a line with an insertion of the same T-DNA, but where no gene is disrupted was used as control. This line and all other T-DNA insertional mutants from the same collection we have tested are resistant to kanamycin 50 mg/l. In this experiment, all seedlings developed long roots in the absence of kanamycin, but in the presence of kanamycin, *wbc19* mutants exhibited their characteristic stunted root growth, although their leaves remained green.

Sequencing libraries were prepared from the seedlings and resulted in an average of 2.3 million reads per library uniquely mapping to the *Arabidopsis* genome (**Table S2**). The differential expression of genes was analyzed using Cuffdiff and deemed significant when p<0.001 and two fold differences were observed. To validate the RNA-seq results, 11 genes were randomly selected among those with a GO classification of interest as listed in Tables 1–6. Their transcript levels were examined by Real-Time PCR under all 4 experimental conditions and 3 sets of comparisons were made (control vs. control + kanamycin; control vs. mutant; control + kanamycin vs. mutant + kanamycin). The overlap between the 3 sets of comparisons from Real-Time data and RNA-seq data was examined. For the total of 30 comparisons where statistical significance could be assessed, a good overlap between Real-Time results and RNA-seq results was observed. For the 13 instances where significant differences were found in the RNA-seq experiment, 11 (84.6%) were also found to be differentially expressed in the Real-Time PCR assays (**File S1**). Thus the false positive discovery rate was at 15.4%. Among the 17 comparisons that were found non-significant in the RNA-seq experiment, 14 (82.3%) remained non-significant when Real-Time PCR assays were done. Thus the false negative rate is estimated at 17.6%. Overall the Real-Time PCR results validate the RNA-seq data although the amplitude of the observed differences were more modest. For Real-Time PCR assays, the highest fold difference was observed for FERRIC REDUCTION OXIDASE 6 (FRO6) where five times more transcripts are expressed in the mutant seedlings exposed to kanamycin compared to the control.

### Transcriptional response of control Arabidopsis seedlings to kanamycin

Although control plants exposed to kanamycin were visually asymptomatic, a total of 116 genes were differentially regulated, of which 88 were upregulated and 28 were downregulated (**File S2**). Examination of over-represented categories (**Figure S1**) indicated that photosynthesis light reaction components were more frequent among the differentially regulated transcripts than might be expected by chance. Further detailed analysis of the GO classification of differentially expressed genes allowed us to identify three overlapping sets of transcripts, given our interest in metal homeostasis. The first set is represented by genes with a direct or indirect role in photosynthesis. For example, increased transcripts of the P subunit of photosystem I (PSI-P) and that of the chlorophyll a/b-binding protein 2 (CAB2), a component of the light harvesting complex associated with photosystem II (PSII) denote changes taking place in both photosystems (**Table 1**). The upregulation of both transcripts was confirmed in Real-Time PCR assays. These changes are accompanied by the upregulation of ALBINO3 which is essential for the efficient assembly of photosystem II [22] as well as DEGP PROTEASE 7 (DegP7) which is involved in the repair of photodamaged PSII via cleavage of its D1 protein [23]. In addition the differential regulation of a number of plastid genes involved in transcription and translation (plastid transcriptionally active 6, Ts translation elongation factor, chloroplastic ribosomal protein L9) further highlight changes taking place in plastids.

A second set of transcripts is represented by redox enzymes. Two enzymes enabling the exchange of electrons with ferredoxins were differentially regulated. The mitochondrial ferredoxin reductase (AtMFDR) was found to be upregulated which was confirmed in Real-Time PCR assays. AtMFDR enables the transfer of electron from NADP(H) to ferredoxin most likely for Fe-S cluster assembly and biotin synthesis in the mitochondria [24], [25]. On the other hand, NAD(P)H dehydrogenase C1(NDC1) was downregulated. NDC1 can localize to both mitochondria and chloroplasts [26], [27], [28] and was shown to reduce the pool of plastoquinone (PQ) in plastoglobules, thereby playing a role in the synthesis of prenylquinones such as vitamin K1 [29]. These changes suggest the presence of alternative pathways of electron flow as these enzymes were shown to have variable metabolic roles. More specifically, biosynthetic pathways in the chloroplast appear to be reduced while biosynthetic pathways in the mitochondria especially Fe-S cluster containing proteins are promoted. Taken together these changes likely reflect an adaptation of the photosynthetic apparatus and metabolic fluxes upon plant exposure to kanamycin.

A third set of differentially expressed genes encode for metal binding proteins (**Table 2**). Among these, the best characterized gene is the Magnesium transporter MGT4 (MRS2-3). Our Real-Time PCR assays also confirmed the upregulation of MGT4 transcripts. MGT4 was initially identified as a candidate locus in a search for quantitative trait loci affecting seed concentrations of Mg^2+^ [30] and later confirmed to transport Mg^2+^ [31]. MRS2-3 could complement yeast deficient in Mg^2+^ transport but direct uptake was not found to be considerably higher than the background level in the mutant. It was therefore suggested that MRS2-3 may act as a slow transporter for Mg^2+^ requiring long time intervals for achieving homeostasis and thus represent a high affinity Mg^2+^ transporter. Further, a number of genes encoding for copper binding proteins were found to be differentially regulated, including the gene encoding for LACCASE 5 (LAC5). Real-Time PCR assays confirmed significantly higher levels of LAC5 transcripts in control plants exposed to kanamycin and generally lower levels in mutants. LAC5 is a member of the laccase-like multicopper oxidases generally viewed as phenoloxidases enabling the polymerization of lignin. However, members of the family were shown to have a ferroxidase activity and thus LAC5 might play a role in iron metabolism [32], [33]. More specifically, they are thought to function in iron-uptake systems similar to FET3 in *Saccharomyces cerevisiae*. In the FET3 system, the strict coupling of Fe^2+^ oxidation by Fet3p with transport of Fe^3+^ by Ftr1p allows for a high affinity transport of iron [34], [35], [36]. Thus, the upregulation of LAC5 may signify that plants exposed to kanamycin switch to a higher affinity iron transport system. These changes along with the decrease in the expression of heavy metal transport/detoxification superfamily protein and increased transcripts from the molybdenum cofactor sulfurase family suggest that plants exposed to kanamycin alter their uptake of metals.

### Differences between control plants and wbc19 knockout mutants

In the absence of kanamycin exposure, no visual differences were observed between control and *wbc19* mutant plants, however the RNA-seq analysis revealed significant differences, with 26 genes being downregulated and 69 genes upregulated in the mutant. These changes were subtle as minor effects on the over-representation of the various functional categories were observed (**Figure S2**). The detailed analysis of GO classifications, however, indicated that a number of transcripts are again found to encode for photosynthesis and oxidoreduction related proteins (**Table 3**). In mutant plants, transcripts of one isoform of the PsbP protein (At1g76450) are downregulated while those of its most similar isoform (At3g05410) are upregulated. PsbP is one of extrinsic proteins on the luminal side of PSII involved in the oxidation of water to molecular oxygen by using light energy. The reaction is catalized by a cluster of three inorganic ions, manganese (Mn), calcium (Ca), and chloride (Cl) and PsbP participates in the regulation of the unique redox environment of this inorganic catalytic center (Debus, 2000). Specifically, PsbP is involved in Ca^2+^ and Cl^−^ retention in PSII as well as the protection of the Mn cluster from a wide range of small (H_2_O_2_, NH_2_NH_2_, NH_2_OH, Fe^2+^) and bulky (benzidine, hydroquinone) reductants [37], [38]; and is essential for normal PSII function [39], [40]. The observed shift from one isoform of PsbP to another in *wbc19* mutants suggests an adaptation of PSII to a new ionic or redox environment.

Ferredoxin 3 (Fd3) is expressed at a much lower level in *wbc19* mutants compared to control plants, a finding that was confirmed in Real-Time PCR assays. While ferredoxins primarily contribute to photosynthesis by shuttling electrons from photosystem I (PSI) to the enzyme ferredoxin:NADP^+^ oxidoreductase (FNR) for reduction of NADP^+^ and eventually provide reducing power for CO_2_ assimilation, they can also contribute to a wide range of metabolic reactions such as nitrogen assimilation [41]. Fd3 is a root type or heterotrophic isoform of ferredoxin [42]. Its redox potential favors electron transfer from NADPH to ferredoxin in a predominantly heterotrophic pathway. A decreased expression of Fd3 in mutants once again suggests changes in metabolic fluxes.

When examining transcripts of metal binding proteins differentially expressed in the mutant, six out of eight encode for zinc binding proteins (**Table 4**). All six transcripts are upregulated in *wbc19* mutant seedlings compared to control seedlings. Most notably, the expression of ZINC TRANSPORTER 1 PRECURSOR (ZIP1) was increased. ZIP1 is a highly specific zinc transporter. It can functionally complement the zrt1/zrt2 yeast double mutant deficient in zinc transport but not the fet3/fet4 mutant indicating that it does not transport iron [43]. Although it is normally expressed in roots and induced upon zinc deficiency [43], it can be activated in shoots in IRT1 mutants which are severely iron and zinc deprived [44]. This upregulation of ZIP1 in mutants suggested an altered Zn homeostasis, similar to Zn deficiency responses.

Additionally, transcripts of a cytochrome B561 are also increased in mutants compared to control seedlings. Cytochrome B561s are typically membrane proteins that catalize a transmembrane electron transport from ascorbate to an extracellular substrate. Although the first well-charaterized cytochrome B561 from chromaffin granules of neuroendocrine tissues reduced intra-vesicular ascorbic acid [45] many cytochrome B561 proteins were later found to have a ferric and cupric reductase activity [46], [47], [48], [49]. The upregulation of cytochrome B561 transcripts in mutants suggested an altered iron and or copper homeostasis.

Overall the comparison of transcripts from *wbc19* mutants and control seedling suggest differences in the architecture of their photosynthetic apparatus, their metabolic fluxes and their metal uptake. These differences are observed while seedlings were grown in the absence of kanamycin, indicating that WBC19 has a physiological role under normal conditions and therefore one that is not strictly adaptive upon exposure to kanamycin.

### Effect of WBC19 on the response to kanamycin

To examine how the lack of WBC19 affects responses to kanamycin, we compared transcript abundance between control plants exposed to kanamycin and mutant plants exposed to kanamycin. We found that 38 genes were upregulated and 98 genes were downregulated in the mutant. The functional categories ‘photosystem’, ‘metal handling’ and ‘transport’ were found to be over-represented (**Figure S3**). Twenty-two of the differentially expressed genes were found to be significantly upregulated in the controls upon exposure to kanamycin but failed to be upregulated in the mutants exposed to kanamycin (**Figure S4**). Most notably, LHCb1.1 and the Magnesium transporter MGT4 failed to be upregulated (**Table 5**). Conversely, transcripts of genes such as the Heavy Metal Transporter and NAD(P)H dehydrogenase C1 (NDC1) that were significantly downregulated in the control upon kanamycin exposure, failed to be downregulated in the mutant. Thus mutant plants did not exhibit the signature responses of the control plants. Instead, among genes encoding for photosynthesis related proteins, we observe a reduced amount of transcripts of PHOTOSYSTEM II REACTION CENTER PROTEIN D2 (PSBD). PSII reaction center proteins D1 and D2 are two chlorophyll binding proteins at the core of the site for oxygen evolution. Under conditions of high light and abiotic stress D1 and D2 are photodamaged and repair consists of disassembly of PSII, synthesis of the proteins and reassembly [50]. The low levels of PSII reaction center protein D2 transcripts detected in mutants exposed to kanamycin suggests a lower rate of PSII repair. In addition, the upregulation of the O subunit of PLASTOQUINONE DEHYDROGENASE COMPLEX (NDH-O) suggests increased cyclic electron flow around PSI. The NDH complex catalyzes the transfer of electrons from ferredoxin and NADH back to plastoquinone [51], [52]. This role of the NDH complex is not crucial under normal conditions as mutants do not exhibit a phenotype, but appears important under conditions of stress [53], [54], [55], [56], [57], [58], [59]. Thus, the response of mutant seedlings to kanamycin suggests an adaptation of photosynthetic apparatus to stress, with a disassembly of PSII and cyclic electron flow around PSI.

When transcripts for metal binding proteins were examined, a total of 20 genes were found to be differentially expressed between control and mutant seedlings exposed to kanamycin (**Table 6**). The majority are downregulated, however the expression of FRO6 is increased by about 5 fold in the mutant exposed to kanamycin. FRO6 is a ferric reductase found in the plasma membrane of mesophyll cells in the shoots [60], [61]. Its expression is light inducible and is thought to coordinate iron uptake with photosynthetic activity. The increase of FRO6 transcripts in mutant seedlings exposed to kanamycin is accompanied by significantly lower levels of NICOTIANAMINE SYNTHASE 1 (NAS1) transcripts and for both genes, Real-Time PCR results confirmed RNA-seq findings. NAS1 enables the synthesis of nicotianamine (NA), a low molecular weight chelator of transition metal cations, including Fe^2+^, Fe^3+^, Cu and Zn [62], [63], [64]. NA facilitates the transport of Fe from the phloem to sink organs via YELLOW-STRIPE1-LIKE (YSL) transporters [65], [66]. These findings suggest iron deficiency in leaves and decreased mobilization of metals via the phloem.

### Plant Metal Content

**Figure 1:**
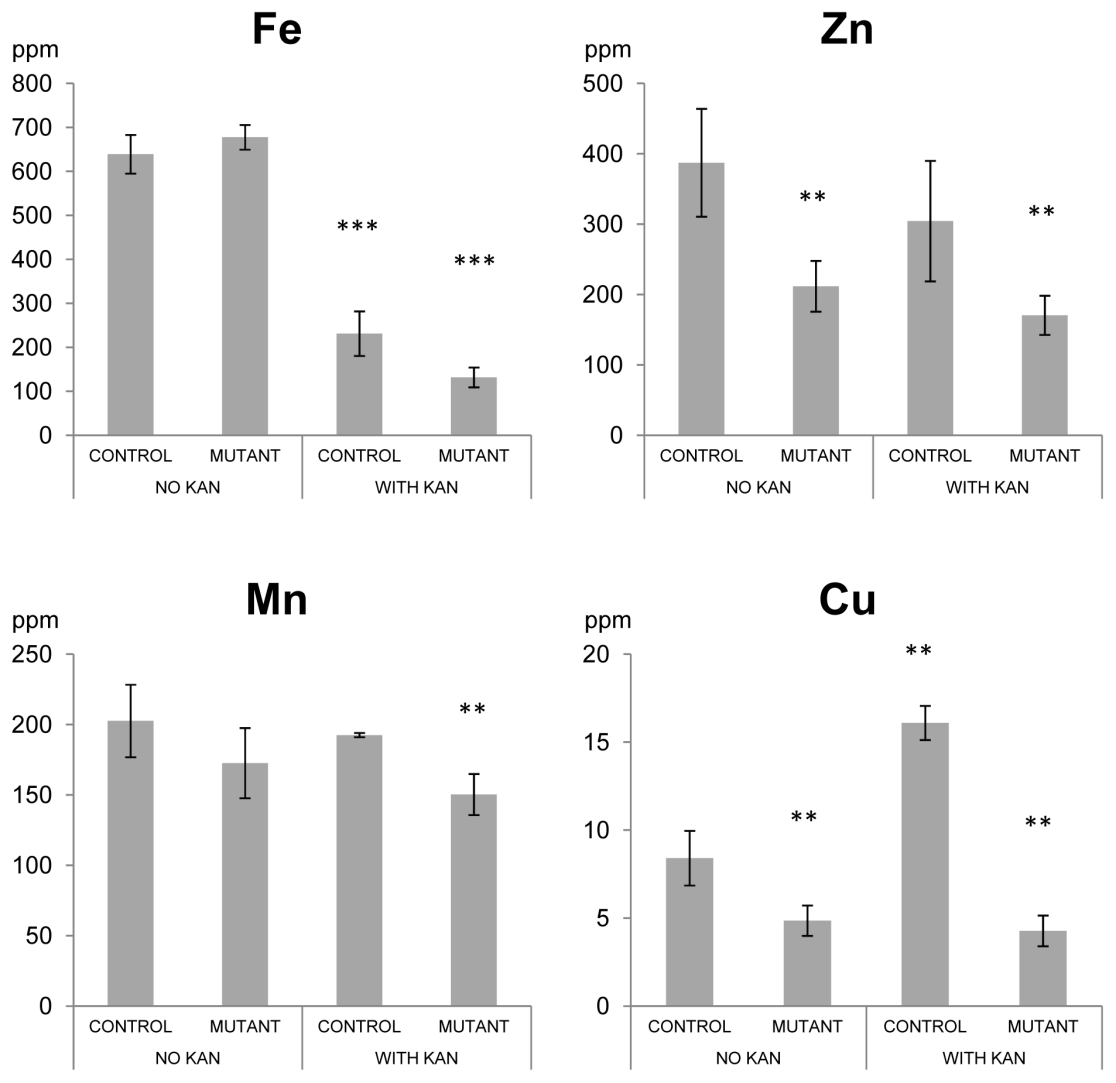
Metal content of seedlings grown in the presence or absence of kanamycin (50mg/l). Iron, zinc, manganese and copper contents from control and *wbc19* mutant seedlings were measured by ICP-MS five days after germination and shown in parts per million (ppm). Error bars represent±standard deviation from 3 biological replicates. **, *** indicate significant difference with the control on media with no kanamycin at P<0.01 and 0.001 respectively (*t*- test).

The RNA-seq results supported our hypothesis that plants’ responses to the antibiotic kanamycin would affect metal homeostasis. Mutation of the kanamycin resistance gene WBC19 further indicated metal uptake deficiencies. We therefore quantified the Fe, Zn, Mn and Cu content of control and mutant plants grown in the presence or absence of kanamycin. Exposure to kanamycin resulted in the reduction of iron content in control seedlings by approximately 60% (**Figure 1**). The reduction in iron content was accompanied by a significant increase in the Cu content by about 50%. As predicted, these results indicate that a major response of plants exposed to kanamycin is to limit their Fe uptake. This in turn may result in secondary effects on the uptake of other metals.

When metal levels of control and *wbc19* mutant plants grown in the absence of kanamycin are compared, significant differences were observed for Zn and Cu. Mutant plants had about 50% less Zn and Cu than control plants while Fe and Mn levels remained unaffected. These results suggested that, WBC19 plays a role in metal uptake even in the absence of kanamycin. Surprisingly, it was not the Fe content that was altered, but that of Zn and Cu.

In mutant plants exposed to kanamycin, Fe levels were even lower than in control seedlings; Cu and Zn levels reach their lowest points and Mn levels reach significantly lower concentrations. Thus mutants exhibit the same response as control plants in reducing their Fe uptake and their overall lower metal content is exacerbated by the exposure to kanamycin. This may be in part due to the stunted roots which further compromised the uptake of nutrients from the media.

### Transcript abundance of iron homeostasis genes

**Figure 2:**
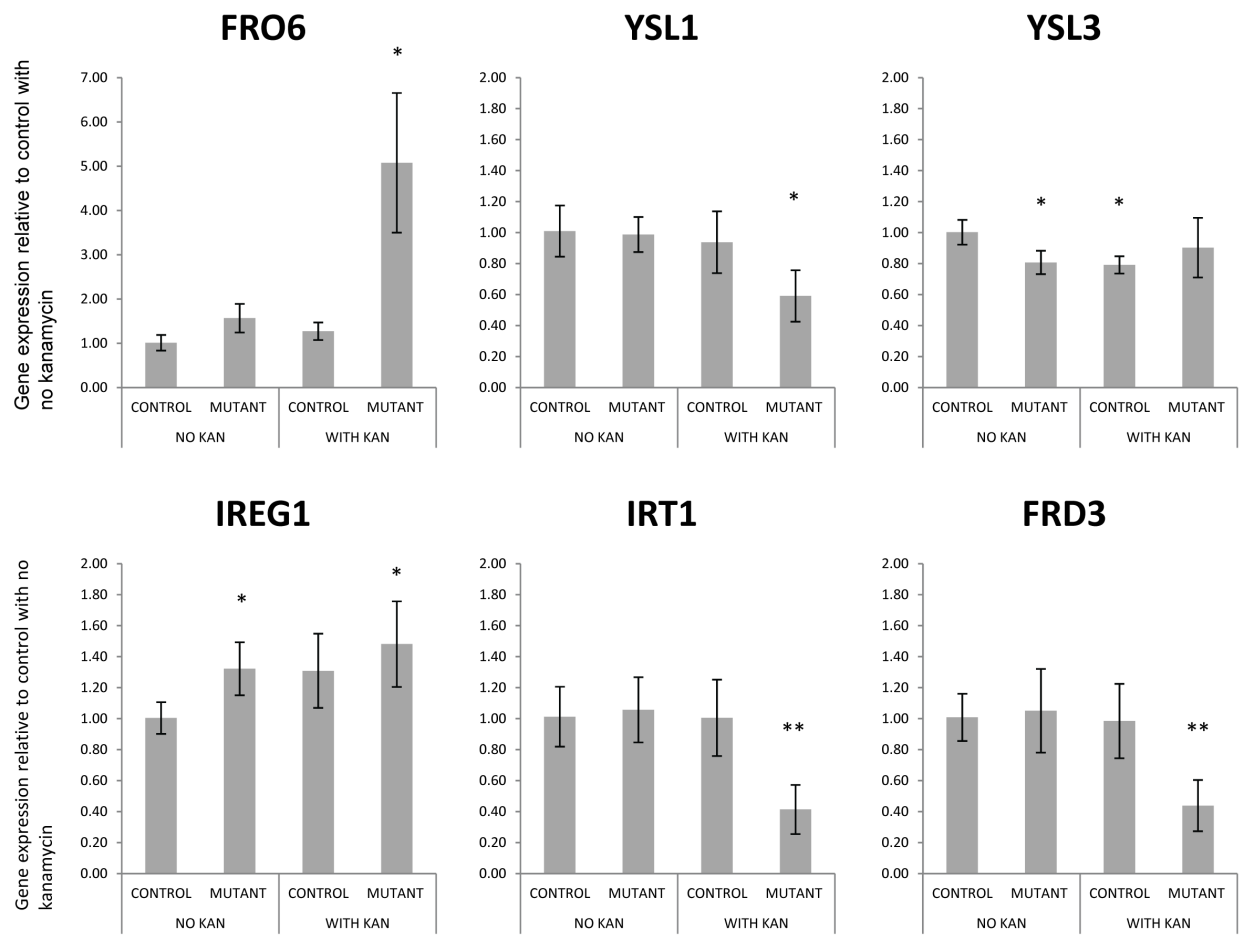
Real-Time RT-PCR analysis of transcript levels of iron homeostasis genes. Multiplexed TaqMan assays were performed for the quantification of iron homeostasis genes as indicated (FAM labeled) and the control, RAD23-3 (VIC labeled) after reverse-transcription of mRNA extracts from control and *wbc19* mutant plants germinated on MS media with or without 50 mg/l kanamycin. Data shown are from 4 biological replicates and 3 technical replicates. Error bars indicate standard deviation.

To further analyze the effect of kanamycin on iron homeostasis, a panel of six well characterized genes was selected for real-time PCR analysis (**Figure 2**). These include genes known to be involved in iron uptake in roots (IRON REGULATED TRANSPOTER 1 (IRT1), FERRIC REDUCTION OXIDASE 2, (FRO2)), iron loading and transport in xylem (IRON REGULATED PROTEIN 1 (IREG1/FPN1), FERRIC REDUCTASE DEFECTIVE 3 (FRD3)), iron unloading and remobilization (YELLOW STRIPE LIKE 1 and 3(YSL1, YSL3)), iron uptake in mesophyll cells (FRO6). The latter was already found to be upregulated in mutant plants exposed to kanamycin and inclusion of this gene would further serve to confirm results from the transcriptome profiling. When control seedlings were exposed to kanamycin, no significant changes in transcript levels were observed for all genes studied except for a small decrease in transcript levels of YSL3. This finding indicates that despite their low levels iron, seedlings exposed to kanamycin do not induce genes known to be upregulated during iron deficiency. On the other hand, there were no differences in iron content between control and mutant seedlings grown in the absence of kanamycin, but transcript levels of IREG1/FPN1 were found to be significantly different. IREG1/FPN1 transcripts were about 30% higher in mutants and increased further upon kanamycin exposure. IREG1/FPN1 is not responsive to iron but is thought to be involved in iron loading in the xylem [67].

In mutant plants exposed to kanamycin several iron homeostasis genes were found to be differentially regulated. Transcripts of IRT1, FRD3 and YSL1 were significantly less abundant. This likely reflected the fact that mutant seedlings had stunted roots as all three transcripts are mainly expressed in roots of seedlings [68], [69], [70]. Transcripts of IREG1 and FRO6 were significantly more abundant when mutant plants were exposed to kanamycin. The results obtained for FRO6 in Real-Time assays corroborated findings from RNA-seq experiments. Mutants exhibited a five-fold increase of FRO6 transcripts, but not controls despite low levels of iron in both lines after germination in the presence of kanamycin. FRO6 encodes for an iron chelate reductase expressed in green tissues [60], [61]. It is was found to be mainly regulated by light rather than iron and thought to coordinate iron needs with photosynthesis. In light of this expression pattern, it is difficult to attribute difference in FRO6 induction between mutant and control plants to the differences in iron content after exposure to kanamycin. Nonetheless, our results show that kanamycin is a potent inducer of FRO6 in *wbc19* mutants. Likewise, a more modest increase in the transcript levels of IREG1 (FPN1) was detected. Previous experiments have shown that IREG1 is mainly expressed in the vasculature of the root and shoot [67] and not iron regulated [71], [72]. It likely effluxes metals from the cytoplasm into the vasculature, allowing movement of metals from root to shoot [67].

### WBC19 is predominantly expressed in vascular tissues

**Figure 3:**
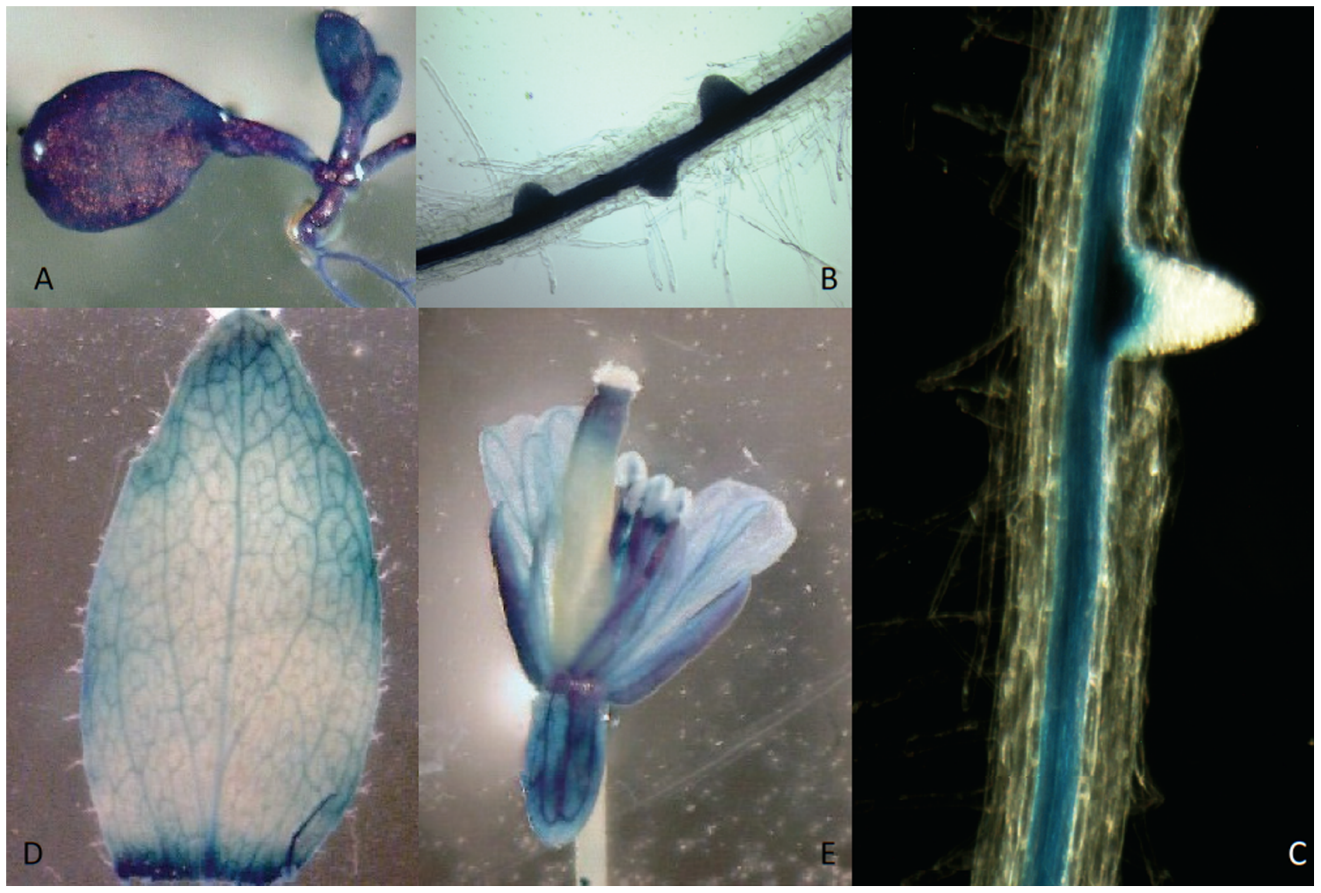
Expression pattern of WBC19. A wbc19 promoter-GUS fusion construct was generated to examine the endogenous expression pattern of wbc19. GUS stained 10 days old seedling showing leaf (A) and roots (B,C); older leaf (D) and flower (E).

To gain further insight on the role of WBC19 in antibiotic resistance, we analyzed the expression pattern of its native promoter. A 2.1 kb DNA fragment upstream of the start codon was cloned in front of a GUS gene and *Arabidopsis* plants containing the constructs assayed for histochemical GUS activity. Strong GUS expression was observed in leaves and stele of 10 day old seedlings (**Figure 3**. A, B, C). Actively dividing cells of the pericycle also showed GUS expression. Once the secondary root emerges, the new meristematic region no longer showed GUS expression. In contrast to what is seen in young leaves, GUS expression in older leaves is restricted to the veins. Similarly in flowers, GUS staining is prominent in the vascular tissue of the sepals, petals and anther filaments (**Figure 3** D, E). Overall, GUS expression was predominantly vascular, a pattern characteristic of genes with a role in the long distance transport of nutrients.

## Discussion

Plants are exposed to a variety of antibiotics produced by soil microorganisms. Therefore understanding how plants have adapted to this prevalent, yet less recognized form of stress is important not only at the fundamental level but also for its implications in plant nutrition, productivity and in genetic engineering. In this study we undertook a large-scale analysis of the effect of kanamycin and examined the role of WBC19 in resistance using the RNA-seq technique. In conjunction with metal analysis and expression profiling with a promoter-GUS fusion, important insights were gained on the physiology of plants exposed to kanamycin and the role of WBC19.

### Plant exposure to kanamycin leads to reduced iron uptake

The elemental analysis showed that *Arabidopsis* seedlings exposed to kanamycin reduced their iron uptake by 60%. Chloroplasts represent the major reservoir of iron in plants where it is needed as a component of PSI and PSII and serves as a co-factor in the electron transport chain during the light reactions of photosynthesis. It is also used for the assembly of Fe-S clusters and the synthesis of chlorophyll [73], [74]. Iron deficiency primarily affects PSI function, probably because of its high requirement for iron (12 Fe atoms per PSI unit) [75]. In turn, the decline in PSI function affects electron transport from PSII to PSI, leading to the generation of reactive oxygen species and photo-oxidative damage. Adaptive responses vary in different organisms but generally involve the dissipation of light energy and optimization of photosynthetic function [75], [76], [77], [78]. In plants, the LHCII plays a critical role in the dissipation of light energy [79] and specific isoforms of LHCII subunits were shown to be induced by iron deficiency [78].

In our experiments, the increase of transcripts encoding for components of PSI and PSII (PSI-P and CAB2/LHCB1.1) and the repair of photodamaged PSII (ALBINO3 and DegP7) captured the adaptation of the photosynthetic apparatus to kanamycin and emulated a response to reduced iron. Overall, the observed changes were limited considering the large reduction in iron content. It is possible that a limited response is observed at the transcript level but more significant changes might take place at the protein level. Previous studies in *Chlamydomonas reinhardtii* have revealed that protein levels of many PS I components decrease under iron limitation [77], but changes in the transcript levels of these proteins were not detected [75]. Thus transcript profiling might only provide a partial portrayal of all the changes that are taking place upon plant exposure to kanamycin.

Prior to its arrival in chloroplasts, iron is acquired from the soil and mobilized to the leaves. Iron acquisition in roots, especially in response to iron deficiency has been well characterized. In *Arabidopsis*, Fe^3+^ in the soil is typically reduced to Fe^2+^ by a ferric oxidase reductase (FRO2) found in root epidermal cells. It is then taken-up by the iron regulated transporter IRT1 that belongs to the ZIP family of metal transporters [70], [71], [80], [81]. Iron moves symplastically in root cells and is loaded into the xylem by IRON REGULATED1 (IREG1/FPN1) [67]. Concomitant loading of citrate by FERRIC REDUCTASE DEFECTIVE3 (FRD3) allows the long-distance transport of iron as an Fe^3+^-citrate chelate [68], [82], [83]. Iron is also mobilized in the phloem as an Fe^2+^-nicotinamine chelate where it can be transported by members of the YSL family of transporters [65], [66], [69], [84]. We examined transcript levels of these genes involved in iron acquisition and mobilization using TaqMan real-time PCR assays. Our results indicated that transcript levels of most genes were not affected by exposure to kanamycin in control plants. This observation indicated that the reduction in iron content was not accompanied by changes resembling iron deficiency responses. Thus, the physiological state induced by kanamycin is distinct from that caused by iron deficiency alone.

Nonetheless, control plants exposed to kanamycin appear to be upregulating a high-affinity iron transport system represented by LAC5. In *Chlamydomonas reinhardtii* a similar multicopper oxidase, FOX1, involved in high-affinity iron uptake is dramatically upregulated under iron limited conditions. FOX1 is upregulated before growth of cells is inhibited such that it used as a sentinel marker in iron deficiency studies [85], [86]. Similarly LAC5 could represents a high-affinity transporter upregulated in response to reduced levels of iron in control plants exposed to kanamycin. The upregulation of LAC5 and transcripts of two other copper binding proteins (SKS17, MGE1) in control plants exposed to kanamycin was concomitant with an increase of the copper content of seedlings. Copper is known to play a role in iron homeostasis as a cofactor in multicopper oxidases such as FOX1, FET3 and caeruploplasmin responsible for high-affinity iron transport in green algae, yeast and humans [34], [35], [36], [85], [86], [87]. More recently copper was also shown to play a role in iron homeostasis in *Arabidopsis*, but specific copper dependent oxidases have not identified [88], [89]. Under our experimental conditions control plants exposed to kanamycin significantly increased their copper uptake. Aside from possibly participating in a high-affinity iron uptake system, increased copper levels may help mitigate oxidative stress under low iron. An increase in copper uptake is generally observed in plants grown under iron limiting conditions and is thought to represent an adaptive response to minimize oxidative stress damage by supplying Cu to Cu Superoxide Dismutases (CuSODs) and compensate for the lack of functional FeSODs [89]. Overall, based on the observed transcriptional changes in control plants exposed to kanamycin, the reduction in iron content could either be a direct consequence of the stress caused by kanamycin on choloroplasts, but could also represent, at least partially, an adaptive response to limit iron uptake.

### WBC19 and zinc homeostasis

Important insights on the role of WBC19 were gained from the comparison of control and mutant plants in the absence of kanamycin. *wbc19* mutants contain about half the amount of zinc and copper compared to control plants suggesting that WBC19 plays an important role in zinc and copper uptake under normal conditions. The low levels of zinc in mutants were accompanied by increased expression of the zinc transporter ZIP1 as well as a number of zinc binding proteins. We sought to confirm increased expression of ZIP1 by real-time RT-PCR, however adequate custom or predesigned primer and probe sets could not be generated for our Taq-Man assays. This was in part due to the fact that ZIP1 transcripts overlapped with ALIS1 transcripts. Zinc is the second most abundant microelement found in plants. In *Arabidopdsis*, zinc is initially taken-up by IRT1 in root epidermal cells along with iron and other metals. Once in the root symplast, it can either be loaded into the xylem mainly by two P-type ATPases, HEAVY METAL ATPASE 2 and 4 (HMA2 and HMA4) [90], [91], [92] sequestered in root vacuoles via two transporters of the cation diffusion facilitator family, the METAL TOLERANCE PROTEIN 1 and 3 (MTP1 and MTP3) [93], [94], [95] or extruded by PLANT CADMIUM RESISTANCE 2 (PCR2) from root epidermal cells under conditions of excess zinc [96]. The presence, transport and compartmentalization of zinc chelators especially nicotianamine (NA) also play an important role in the uptake and distribution of zinc [64], [97], [98], [99].

It is not clear how WBC19 contributes to zinc homeostasis. The phenotype observed for *wbc19* mutants bares similarities with the phenotypes of the *hma2 hma4* double mutants and *hma4* single mutants [90] but also the ZINC-INDUCED FACILITATOR1 (ZIF1) overexpressing lines [64] and the NICOTIANAMINE SYNTHASE (NAS) mutants [99]. ZIF1 is an NA transporter that localizes to the vacuolar membranes. Its overexpression leads to the enhanced sequestration of zinc in root cell vacuoles and therefore a decrease in the mobilization of zinc from roots to shoots. The dramatic effect of ZIF1 on zinc mobilization, but limited effect on iron mobilization is explained by the differences in the stability of Fe-NA and Zn-NA complexes in the vacuole [64], [97], [100]. Fe-NA complexes are not stable at vacuolar pH, whereas Zn-NA complexes are. Similarly, *nas* mutants are deficient in their ability to mobilize zinc from root to shoot but not iron [99]. Although NA has a high affinity for copper, differences in the mobilization of Cu were not observed with ZIF1 overexpressing lines [64] nor *nas* mutants [99]. Our study however also points to a decrease in the uptake of copper by *wbc19* mutants. Given the phenotypic similarities between *wbc19* mutants with *hma2 hma4* double mutants and *hma4* single on one hand and ZIF1 overexpressors and *nas* mutants on the other, WBC19 could have a function in zinc or NA movement. Since WBC19 has a role in antibiotic resistance, a function linked to NA movement is more likely.

NA allows the long-distance transport of iron in the phloem and probably that of zinc and copper in both the xylem and phloem [66], [100], [101]. NA is a non proteinogenic amino acid synthesized by a condensation reaction of three molecules of *S*-adenosyl-Met by nicotianamine synthase (NAS) [102]. Four NAS genes are encoded in the *Arabidopsis* genome and their transcript levels are upregulated in response to iron, zinc and copper deficiency [103], [104]. With the optimal spacing of its six functional groups, the structure of NA allows octahedral coordination and formation of chelate rings, making it a high affinity chelator of several metals [101]. It is also thought that the structure of NA or metal-NA complexes bears similarities with that of aminoglycoside antibiotics such that they can move through the same transporters [6], [8]. In *wbc19* mutants, defects in zinc deficiency are coupled to kanamycin sensitivity. One possible explanation is that mutants have increased expression of NA transporters for xylem loading. In turn this leads to increased transport of kanamycin. Conversely, when WBC19 is functional, lower levels of NA transporters are present and thus result in decreased kanamycin movement. Our analysis of the expression pattern of WBC19 further support the notion that it might have a role in long-distance transport as expression was predominantly vascular. Future studies will examine the relationship between NA, zinc and WBC19.

## Conclusion

Plants mine the soil to obtain essential nutrients and in so doing can take-up antibiotics produced by soil microorganisms. Our study supports the view that chloroplasts are an important target of aminoglycosides as several transcripts of the photosynthetic apparatus were differentially expressed upon seedling exposure to kanamycin. Another consequence of the exposure to kanamycin is a significant decrease in iron content. It is unknown whether this is due to a decreased requirement for iron or an adaptive response to limit iron uptake and possibly restrict kanamycin movement. We also observed notable differences between control and *wbc19* mutants. The fact that mutant seedlings upregulate FRO6 while control seedlings do not highlights a major difference in the way kanamycin affects iron homeostasis in the two lines. Hence, aside from lower levels of zinc observed in mutant, more subtle differences with regard to iron homeostasis are affected by WBC19.

## Supporting Information

Figure S1 **Over-represented functional categories for transcripts differentially expressed upon germination of control seedlings on media with kanamycin.** The normalized representation relative to the frequency of group members in the *Arabidopsis* genome (± bootstrap StdDev) is shown. Significantly overrepresented groups (P<0.05) are in bold.
(JPG)

Figure S2 **Over-represented functional categories for transcripts differentially expressed in** ***wbc19*** **mutant seedlings.** The normalized representation relative to the frequency of group members in the *Arabidopsis* genome (±bootstrap StdDev) is shown. Significantly overrepresented groups (P<0.05) are in bold.
(JPG)

Figure S3 **Over-represented functional categories for transcripts differentially expressed when comparing control and** ***wbc19*** **mutant seedlings germinating on media with kanamycin.** The normalized representation relative to the frequency of group members in the *Arabidopsis* genome (±bootstrap StdDev) is shown. Significantly overrepresented groups (P<0.05) are in bold.
(JPG)

Figure S4 **Venn Diagram showing the overlap between genes found to be significantly up or downregulated in the control exposed to kanamycin compared to the control, and genes differentially expressed between control and mutant plants exposed to kanamycin (fold change>2; P<0.001).**
(JPG)

Table S1 Pre-Designed TaqMan Assays from Life Technologies used in multiplex Real-Time PCRs for the quantification of various iron homeostasis genes (FAM labeled) and the control, RAD23-3 (VIC labeled).
(DOCX)

Table S2 Mapping efficiency of reads obtained for each RNA-seq library. An average of 2.3 million reads per library uniquely mapped to the Arabidopsis genome.
(DOCX)

File S1 **Real-Time PCR validation of RNA-seq data on a random selection of eleven genes across the four treatment conditions.**
(XLSX)

File S2 **RNA-seq analysis and differentially expressed genes.** The columns were color coded as follows: yellow: gene descriptions retrieved from TAIR, blue: CuffDiff output when comparing control no kan and mutant no kan samples, pink: CuffDiff output when comparing control + kan and mutant + kan samples, green: CuffDiff output when comparing control no kan and control + kan samples, orange: CuffDiff output when comparing mutant no kan and mutant + kan samples.
(XLSX)
