# Supplementary figures and images for: RNA-Seq Analysis of the Effect of Kanamycin and the ABC Transporter AtWBC19 on Arabidopsis thaliana Seedlings Reveals Changes in Metal Content
Source: PLoS One. 2014 Oct 13;9(10):e109310. doi: 10.1371/journal.pone.0109310 (PMC4195610; doi:10.1371/journal.pone.0109310)

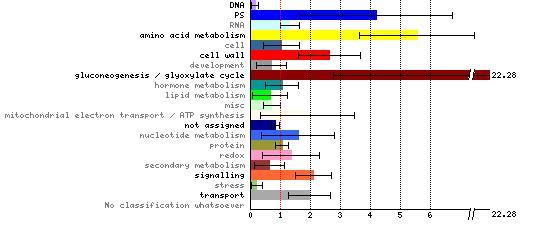

Supplement: Figure S1 — Over-represented functional categories for transcripts differentially expressed upon germination of control seedlings on media with kanamycin. The normalized representation relative to the frequency of group members in the Arabidopsis genome (± bootstrap StdDev) is shown. Significantly overrepresented groups (P<0.05) are in bold. (JPG) [file pone.0109310.s001.jpg]

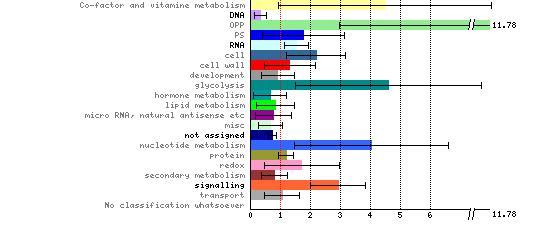

Supplement: Figure S2 — Over-represented functional categories for transcripts differentially expressed in wbc19 mutant seedlings. The normalized representation relative to the frequency of group members in the Arabidopsis genome (±bootstrap StdDev) is shown. Significantly overrepresented groups (P<0.05) are in bold. (JPG) [file pone.0109310.s002.jpg]

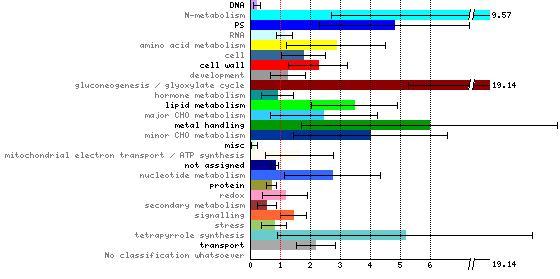

Supplement: Figure S3 — Over-represented functional categories for transcripts differentially expressed when comparing control and wbc19 mutant seedlings germinating on media with kanamycin. The normalized representation relative to the frequency of group members in the Arabidopsis genome (±bootstrap StdDev) is shown. Significantly overrepresented groups (P<0.05) are in bold. (JPG) [file pone.0109310.s003.jpg]

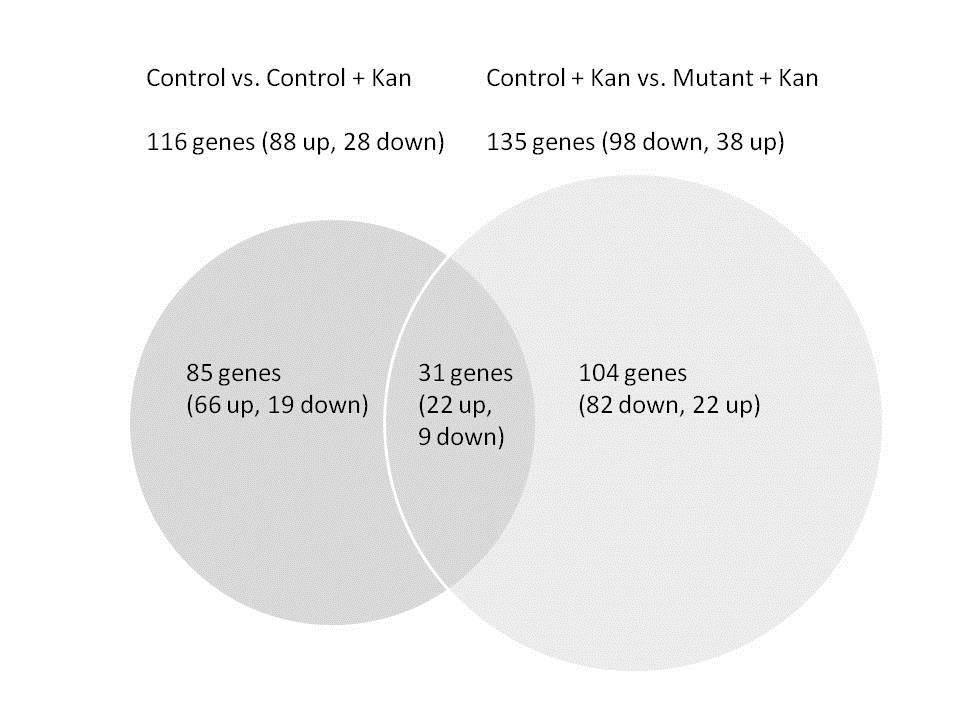

Supplement: Figure S4 — Venn Diagram showing the overlap between genes found to be significantly up or downregulated in the control exposed to kanamycin compared to the control, and genes differentially expressed between control and mutant plants exposed to kanamycin (fold change>2; P<0.001). (JPG) [file pone.0109310.s004.jpg]
