# Supplementary material for: RNA-Seq Analysis of the Effect of Kanamycin and the ABC Transporter AtWBC19 on Arabidopsis thaliana Seedlings Reveals Changes in Metal Content
Source: PLoS One. 2014 Oct 13;9(10):e109310. doi: 10.1371/journal.pone.0109310 (PMC4195610; doi:10.1371/journal.pone.0109310)
Supplement: Table S1 — Pre-Designed TaqMan Assays from Life Technologies used in multiplex Real-Time PCRs for the quantification of various iron homeostasis genes (FAM labeled) and the control, RAD23-3 (VIC labeled). (DOCX) [file pone.0109310.s005.docx]

**Table S1**. Pre-Designed TaqMan Assays from Life Technologies used in multiplex Real-Time PCRs for the quantification of various iron homeostasis genes (FAM labeled) and the control, RAD23-3 (VIC labeled).

| **Gene** | **Gene ID** | **Description** | **Assay ID** |
| --- | --- | --- | --- |
| RAD23-3 (control) | At3g02540 | Putative DNA repair protein | At02163241_g1 |
| PSI-P | AT2G46820 | Photosystem I P subunit | At02263994_g1 |
| LHCB1.1 | AT1G29920 | Chlorophyll A/B-binding protein | At02168205_s1 |
| LAC5 | AT2G40370 | Laccase 5 | At02325202_g1 |
| PHYB | AT2G18790 | Phytochrome B | At02177335_g1 |
| FD3 | AT2G27510 | Ferredoxin 3 | At02357365_s1 |
| AtMFDR | AT4G32360 | Mitochondrial Ferredoxin reductase  (AtMFDR) | At02248462_g1 |
| PsbP | AT1G76450 | Photosystem II reaction center PsbP family protein | At02287233_g1 |
| mtACP2 | AT1G65290 | Mitochondrial acyl carrier protein 2 | At02219002_m1 |
| MGT4 | AT3G19640 | Magnesium transporter 4 | At02254929_g1 |
| NAS1 | At5g04950 | Nicotianamine synthase 1 | At02181318_s1 |
| FRO6 | At5g49730 | Ferric reduction oxidase 6 | At02318925_g1 |
| YSL1 | At4g24120 | Yellow stripe like 1 | At02237465_g1 |
| YSL3 | At5g53550 | Yellow stripe like 3 | At02321256_g1 |
| FRD3 | AT3G08040 | Ferric reductase defective 3 | At02237261_g1 |
| IREG1 | AT2G38460 | Iron-regulated protein 1 | At02324075_g1 |
| IRT1 | At4g19690 | Iron-regulated transporter | At02164076_gH |
