# Supplementary material for: RNA-Seq Analysis of the Effect of Kanamycin and the ABC Transporter AtWBC19 on Arabidopsis thaliana Seedlings Reveals Changes in Metal Content
Source: PLoS One. 2014 Oct 13;9(10):e109310. doi: 10.1371/journal.pone.0109310 (PMC4195610; doi:10.1371/journal.pone.0109310)
Supplement: Table S2 — Mapping efficiency of reads obtained for each RNA-seq library. An average of 2.3 million reads per library uniquely mapped to the Arabidopsis genome. (DOCX) [file pone.0109310.s006.docx]

**Table S2**. Mapping efficiency of reads obtained for each RNA-seq library. An average of 2.3 million reads per library uniquely mapped to the *Arabidopsis* genome.

|  | mapped reads | unmapped QC passed reads | unmapped QC failed reads | total | percent mapped reads |
| --- | --- | --- | --- | --- | --- |
| S1 | 1,748,875 | 369,142 | 2,448 | 2,120,465 | 82.47601 |
| S2 | 1,624,698 | 352,230 | 886 | 1,977,814 | 82.14615 |
| S6 | 3,167,461 | 1,365,688 | 2,391 | 4,535,540 | 69.83647 |
| S7 | 2,715,250 | 1,199,025 | 2,240 | 3,916,515 | 69.32822 |
